# The impact of mini-invasive right hepatectomy in the setting of living donation: a meta-analysis

**DOI:** 10.1007/s13304-021-01160-x

**Published:** 2021-09-06

**Authors:** Quirino Lai, Francesco Giovanardi, Gianluca Mennini, Giammauro Berardi, Massimo Rossi

**Affiliations:** 1 General Surgery and Organ Transplantation Unit, Department of General and Specialistic Surgery, Umberto I Polyclinic of Rome, Sapienza University of Rome, Viale del Policlinico 155, 00161 Rome, Italy; 2 Center for Advanced Treatment of HBP Diseases, Ageo Central General Hospital, Tokyo, Japan

**Keywords:** Laparoscopic, Living donor right hepatectomy, Living donor liver transplantation, Minimally invasive, Robotic, Hand-assisted, Laparoscopic-assisted

## Abstract

Adult-to-adult living-donor liver transplantation (A2ALDLT) represents a challenging procedure, mainly when the right hepatic lobe is donated. Therefore, especially in Western countries, the medical community still considers it a “risky procedure”. The present meta-analysis investigated the postoperative results reported in donors undergoing right hepatectomy for A2ALDLT through a minimally invasive liver resection (MILR) vs. open liver resection (OLR) approach, with the intent to clarify the hypothesis that the MILR approach should minimize the risks for the donor. A systematic literature search was performed using MEDLINE-PubMed, Cochrane Library, and EMBASE electronic databases. The primary outcome investigated was the complication rate after transplant. Fifteen studies were included (*n* = 2094; MILR = 553 vs. OLR = 1541). The MILR group only merged the statistical relevance in terms of advantage in terms of a lower number of complications (OR = 0.771, 95% CI 0.578–1.028; *P* value = 0.077). Investigating the complications ≥ IIIa according to the Dindo-Clavien classification, the estimated blood loss, and the length of hospital stay, no statistical difference was reported between the two groups. MILR represents a novel and promising approach for improving the results in A2ALDLT. However, no benefits have been reported regarding blood loss, length of stay, and postoperative complications. More extensive experiences are needed to re-evaluate the impact of MILR in right lobe live donation.

## Introduction

The considerable progress made in conventional liver surgery and the experience gained from technical variants of whole liver transplantation consented to develop the first living-donor liver transplantation (LDLT) experiences [1, 2]. However, mainly in the specific setting of adult-to-adult (A2A)LDLT, several concerns were raised in terms of donor safety [3]. These obstacles were successfully overpassed in Asian countries, where the problem of deceased donation shortage was critical due to religious and cultural issues [4]. Therefore, the first series of A2ALDLT were performed using a left or a right hepatic lobe [5, 6].

However, A2ALDLT remains a challenging procedure, mainly when the right hepatic lobe is donated. This datum explains why, especially in Western countries, the medical community still considers the right hemi-liver donation as a “risky procedure” to be performed with caution and under certain conditions [7]. With the intent to minimize the risks of donation, the use of a minimally invasive liver resection (MILR) approach for liver donation has been postulated instead of a standard open liver resection (OLR). Increasing evidence has been reported on the safe use of MILR in several different liver diseases, as clearly stated in the Consensus Conferences of Louisville 2008, Morioka 2014, and Southampton 2017 [8–10]. Recently, International Expert Consensus Guidelines have been published explicitly investigating the impact of MILR in the setting of liver donation [11]. However, some questions still require a definitive answer, mainly in the right lobe MILR for A2ALDLT. With the intent to answer to these questions, we decided to perform a meta-analysis able to investigate the postoperative results reported in donors undergoing right hepatectomy for A2ALDLT through MILR vs. OLR approach. Our hypothesis, derived from the hepatic resection experiences, was that the MILR approach should decrease the risks of post-operative adverse course in the donors.

## Materials and methods

### Research strategy

Systematic research has been carried out on the role of MILR in A2ALDLT. The research strategy was carried out following the guidelines of the “Preferred Reporting Items for Systemic Reviews and Meta-Analysis (PRISMA)” and the PRISMA for abstracts [12].

The specific research question formulated in this study includes the following components of PICO:

Patient: ann individual undergoing right hepatectomy for A2ALDLT;

Intervention: right hepatectomy performed with MILR;

Comparison: right hepatectomy performed with OLR;

Outcome: duration of surgery/intraoperative blood loss/post-operative transaminases peak/any post-operative complication/post-operative Dindo-Clavien complication ≥ IIIa/duration of post-operative hospitalization.

A search was performed through MEDLINE-PubMed, Cochrane Library and EMBASE electronic databases, using the following keywords: “(liver OR hepat*) AND (transplant*) AND (laparosc* OR robot*) AND (donor OR donation)”. Studies published before September 01, 2020 have been evaluated.

### Screening process

The qualitative systematic review included “a priori” research of scientific articles concerning adult patients (age > 18 years). Only articles in the English language were considered. All the studies with a comparative analysis between MILR and standard OLR for A2ALDLT donation were considered eligible. Exclusion criteria in the selection of the articles were: (a) insufficiently detailed articles; (b) reviews; (c) non-clinical studies; (d) expert opinions; (e) letters to the editor; (f) conference summaries; and (g) case reports. When studies coming from the same center were identified, a check for data overlapping was performed. In the case of overlapping, we considered only the study with the most considerable reported experience. Two independent authors (QL and FG) performed the screening process of the articles. During article selection, potential differences were resolved through consensus with a third reviewer (MR).

### Data extraction

After the screening process, the selected articles’ full text was analyzed in detail for data extraction. Two independent authors (QL and FG) performed data extraction and compared the results. During data extraction, potential differences were resolved through consensus with a third reviewer (MR).

The characteristics derived from each study were collected in Tables 1, 2 and 3. The following characteristics were collected: author, year, type of surgical approach, type of incision, conversion in case of minimally invasive approach, age, sex, BMI, duration of surgery, estimated blood loss, complications, rate of complication ≥ IIIa according to Dindo-Clavien classification, postoperative AST peak, postoperative ALT peak, and duration of hospitalization.

**Table 1:** Different types of minimally invasive and incision approach observed in the extracted studies

Author [References]	Center	Year	MILR	Type	Conversion	Incision in MILR	OLR	Incision in OLR
Baker [16]	Chicago, US	2009	33	LA	2	5-cm upper midline	33	J-shaped
Choi [17]	Catholic University Korea Seoul, Korea	2012	20 40	LA Single-port LA	NA	15-cm right subcostal + 3 ports	90	Right subcosal
Nagai [18]	Detroit, US	2012	28	LA	0	10-cm UML	30	J-shaped
Ha [19]	Asan Medical Center Seoul, Korea	2013	20	Hand-assisted	0	8-cm right subcostal + 3 ports 15-cm right subcostal + 1 port	20	10- or 12-cm right subcostal
Makki [20]	Noida, India	2014	26	LA	0	6-cm UML	24	J-shaped
Choi [21]	Seoul National University, Korea	2014	25	Hand-assisted	NA	9-cm right subcostal	484	Mercedes-Benz
Suh [22]	Seoul National University, Korea	2015	14	LA	NA	Transverse	268 147	L-shaped 12- to 18-cm UML
Shen [23]	Sichuan University, China	2016	28	LA	1	10-cm UML	20	UML
Chen [24]	Taipei, Taiwan	2016	13	Robotic	0	Pfannestiel	54	Mercedes-Benz
Kitajima [25]	Kyoto University, Japan	2017	41	LA	0	8-cm UML	39	L-shaped
Kobayashi [26]	Niigata, Japan	2018	11	LA	0	12-cm UML	40	Mercedes-Benz
Lee [27]	Seoul National University, Korea	2019	35	Pure lap	2	Pfannestiel	43	L-shaped
Broering [28]	Riyadh, Saudi Arabia	2020	35	Robotic	0	Pfannestiel	70	J-shaped
Jeong [29]	Samsung Medical Center	2020	123	Pure lap	5	Pfannestiel	123	Mercedes-Benz
Lei [30]	Taipei, Taiwan	2020	61	LA	0	10-cm UML	56	J-shaped

*Ref* reference, *MILR* mini-invasive liver resection, *OLR* open liver resection, *LA* laparoscopic-assisted, *UML* upper midline

**Table 2:** Donor characteristics in the different studies and meta-analysis results

Author [References]	Year	Age years		Male sex		BMI	
		MILR	OLR	MILR	OLR	MILR	OLR
Baker [16]	2009	37.0	39.1	15	13	25.8	25.9
Choi [17]	2012	29.7	36.8	12	58	23.6	23.6
Nagai [18]	2012	34.3	38.6	15	9	24.0	30.1
Ha [19]	2013	25.0	29.0	34	17	23.3	23.6
Makki [20]	2014	27.5	32.4	13	18	24.2	24.4
Choi [21]	2014	25.0	NA	1	NA	21.1	NA
Suh [22]	2015	24.9	34.0	1	206	20.9	23.2
Shen [23]	2016	40.4	38.3	15	13	23.1	21.9
Chen [24]	2016	NA	NA	4	24	21.9	22.7
Kitajima [25]	2017	52.0	50.0	15	18	22.0	21.7
Kobayashi [26]	2018	28.0	46.0	7	24	20.8	21.9
Lee [27]	2019	31.4	35.8	19	21	24.0	23.1
Broering [28]	2020	28.6	26.0	22	46	23.4	23.4
Jeong [29]	2020	30.0	31.0	71	73	NA	NA
Lei [30]	2020	33.4	31.5	24	36	24.3	23.7

Outcome of interest	Study (*n*)	MILR (*n*)	OLR (*n*)	WMD/OR (95% CI)	*P* value	Study heterogeneity		*P* value
						*df*	*I*^2^%	
Donor age (years)	9	305	731	− 0.349 (− 0.609 to − 0.089)	0.008	8	62.208	0.007
Male sex	14	528	1057	0.580 (0.357–0.941)	0.027	13	70.54	< 0.001
BMI	10	340	801	− 0.109 (− 0.408 to 0.189)	0.472	9	75.169	< 0.001

*Ref* reference, *MILR* mini-invasive liver resection, *OLR* open liver resection, *BMI* body mass index, *EBL* estimated blood loss, *NA* not available, *n* number of cases, *WMD* weighted mean difference, *OR* odds ratio, *CI* confidence intervals, *I*^*2*^ Higgins statistic squared

**Table 3:** Postoperative course in the different studies and meta-analysis results

Author [References]	Year	Operative time min		EBL mL		AST peak IU/L		ALT peak IU/L		Complications		Complications DC ≥ IIIa		LOS days	
		MILR	OLR	MILR	OLR	MILR	MILR	MILR	OLR	MILR	OLR	MILR	OLR	MILR	OLR
Baker [16]	2009	265	316	417	550	NA	NA	NA	NA	7	7	0	0	NA	NA
Choi [17]	2012	384	303	870	532	232	232	286	225	12	25	10	20	12	12
Nagai [18]	2012	371	363	212	316	345	345	361	311	6	5	2	2	6	8
Ha T [19]	2013	336	305	290	250	149	149	164	199	1	1	0	0	11	11
Makki [20]	2014	703	675	337	396	262	262	194	220	4	5	1	2	NA	NA
Choi [21]	2014	484	272	308	311	NA	NA	NA	NA	35*	635*	2	18	9	9
Suh [22]	2015	334	276	298	333	177	177	160	143	0	31	0	5	10	9
Shen [23]	2016	386	366	384	417	313	313	352	233	5	1	1	0	7	7
Chen [24]	2016	596	383	169	146	234	234	269	252	1	5	1	1	7	7
Kitajima [25]	2017	431	402	201	313	NA	NA	NA	NA	9	13	0	3	12	12
Kobayashi [26]	2018	475	370	350	480	NA	NA	NA	NA	1	6	1	0	10	11
Lee [27]	2019	434	346	572	559	265	265	285	161	6	14	3	5	10	9
Broering [28]	2020	504	331	250	300	NA	NA	NA	NA	2	12	0	1	5	6
Jeong [29]	2020	335	330	NA	NA	NA	NA	NA	NA	35	33	12	14	9	10
Lei [30]	2020	437	393	298	311	NA	NA	NA	NA	11	15	7	7	13	11

Outcome of interest	Study (*n*)	MILR (*n*)	OLR (*n*)	WMD/OR (95% CI)	*P* value	Study heterogeneity		*P* value
						*df*	*I*^2^%	
Operative time (min)	11	463	924	0.608 (0.095–1.121)	0.020	10	70.54	< 0.001
Estimated blood loss (mL)	10	330	1,215	− 0.129 (− 0.421 to 0.162)	0.384	9	74.032	< 0.001
Post AST peak (IU/L)	7	211	642	0.326 (− 0.051 to 0.703)	0.090	6	76.954	< 0.001
Post ALT peak (IU/L)	7	211	642	0.334 (− 0.041 to 0.710)	0.081	6	79.831	< 0.001
Post complications	14	528	1057	0.771 (0.578–1.028)	0.077	13	0	0.809
Post complications DC ≥ IIIa	14	528	1057	0.837 (0.578–1.278)	0.401	13	0	0.811
Length of hospital stay (d)	7	246	674	− 0.006 (− 0.316 to 0.305)	0.972	6	67.485	0.005

*Ref* reference, *EBL* estimated blood loss, *AST* aspartate aminotransferase, *ALT* alanine aminotransferase, *DC* Dindo-Clavien, *LOS* length of stay, *MILR* mini-invasive liver resection, *OLR* open liver resection, *NA* not available, *n* number of cases, *WMD* weighted mean difference, *OR* odds ratio, *CI* confidence intervals, *I*^*2*^ Higgins statistic squared

*Total number of complications reported instead of the number of donors experiencing a complication

### Quality assessment

The selected studies have been systematically reviewed to identify potential sources of error. The work quality was defined using the Risk of Bias In Non-randomized Studies of Interventions (Robins-I) tool [13].

### Statistical analysis

The meta-analyses were performed using the OpenMetaAnalyst [14]. The continuous variables were expressed as average ± standard deviations. Continuous variables reported as medians in the included studies were transformed into means and standard deviations (https://smcgrath.shinyapps.io/estmeansd/) [15]. In continuous data, the Weighted Mean difference (WMD) was used as a summary measure between the groups. For dichotomous data, the Odds Ratio (OR) was used. In both the measurements, 95% confidence intervals (95% CI) were also reported. The statistical heterogeneity was evaluated with the Higgins statistic squared (*I*^2^). *I*^2^ values were considered indicative of heterogeneity among the studies: low = 0–25%; 26–50% = moderate; ≥ 51% = high. The fixed-effects model was used when low-to-moderate (0–50%) heterogeneity was detected among the studies. The random-effects model was used when high heterogeneity was reported. A *P* value < 0.05 was considered indicative of statistical significance.

## Results

### Characteristics of selected articles

**Fig. 1:**
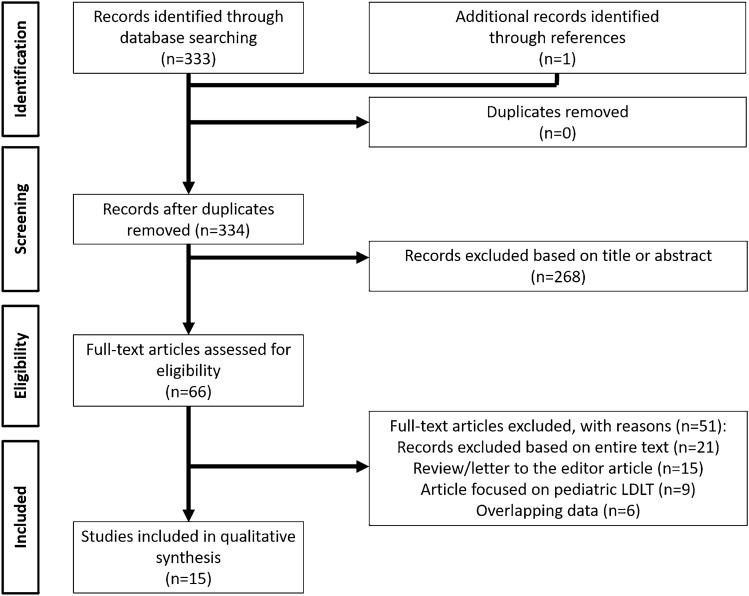
PRISMA chart for papers selection for meta-analysis

The article selection process is explained in Fig. 1. A total of 333 articles was initially identified for screening. A further article was added after a manual search. Two hundred and sixty-eight articles were removed according to their title or abstract evaluation. Of the 66 remaining papers, 21 were excluded after the entire text evaluation. Fifteen articles were excluded from the analysis as reviews, letters to the editor, and commentaries. Nine articles were removed because exclusively dedicated to pediatric LDLT or reporting a mixed adult/pediatric activity. Six articles were further removed because they reported overlapping data. Lastly, 15 studies were selected for a total of 2094 cases investigated. The MILR cases were 553 (26.4%), and the OLR 1,541 (73.6%) [16–30].

**Fig. 2:**
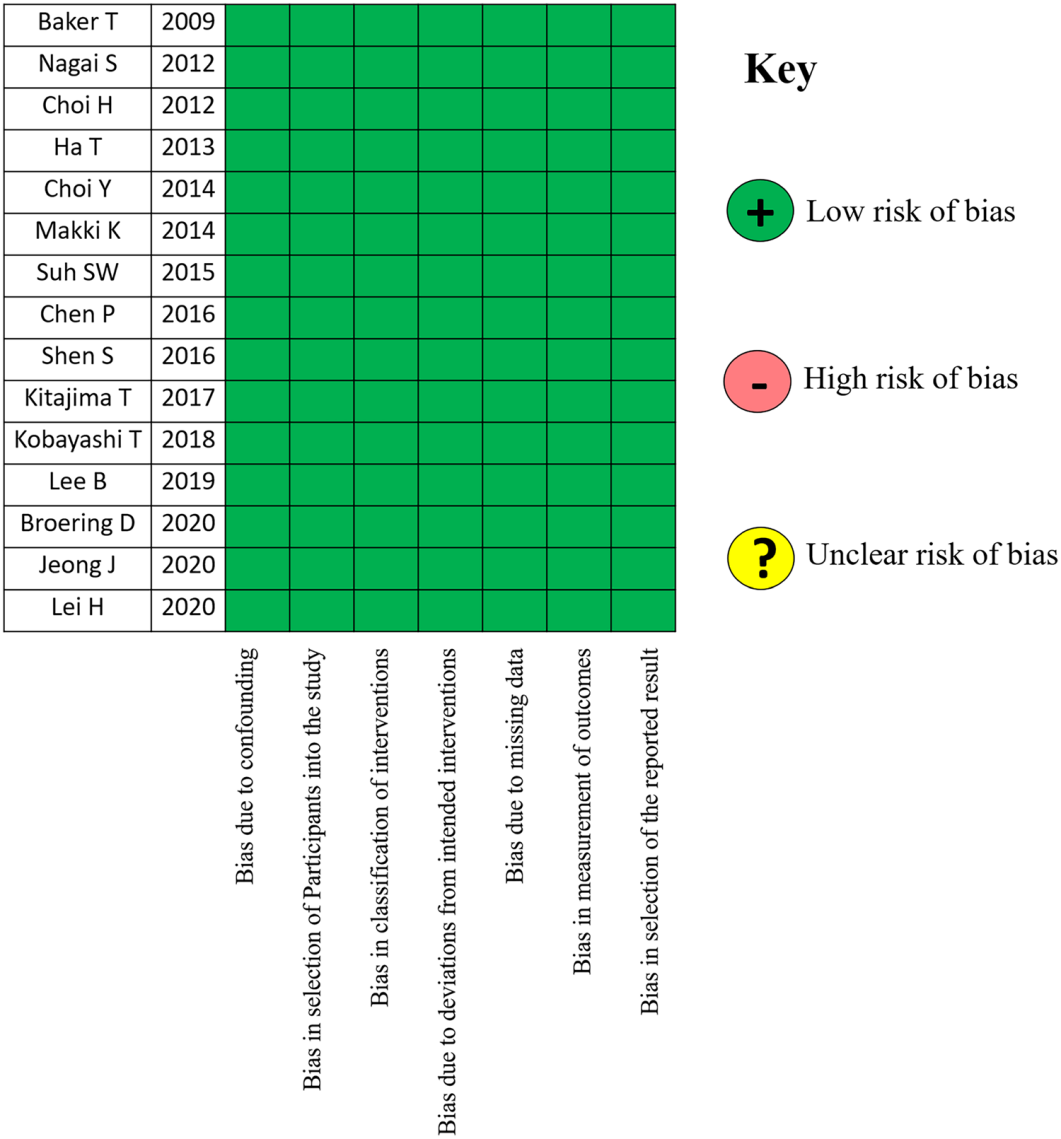
Robins-I for the risk of bias in extracted papers

Regarding the quality of the studies reported, all the articles investigated were retrospective cohort studies with a low risk of bias according to the criteria proposed by Robins-I. No randomized controlled trials were present among the selected studies. Figure 2 shows the overall high quality of the studies identified.

### Surgical techniques

As reported in Table 1, a laparoscopic-assisted approach was reported in nine articles (*n* = 302/553; 54.6%). A hand-assisted technique was performed in two studies (*n* = 45/553; 8.1%). These procedures were classified in the “hybrid” sub-group (*n* = 347/553; 62.7%).

Pure laparoscopic (*n* = 158/553; 28.6%) and robotic (*n* = 48/553; 8.7%) surgery were documented in two articles each. Both these procedures were classified in the “pure laparoscopy” sub-group (*n* = 206/553; 37.3%).

**Fig. 3:**
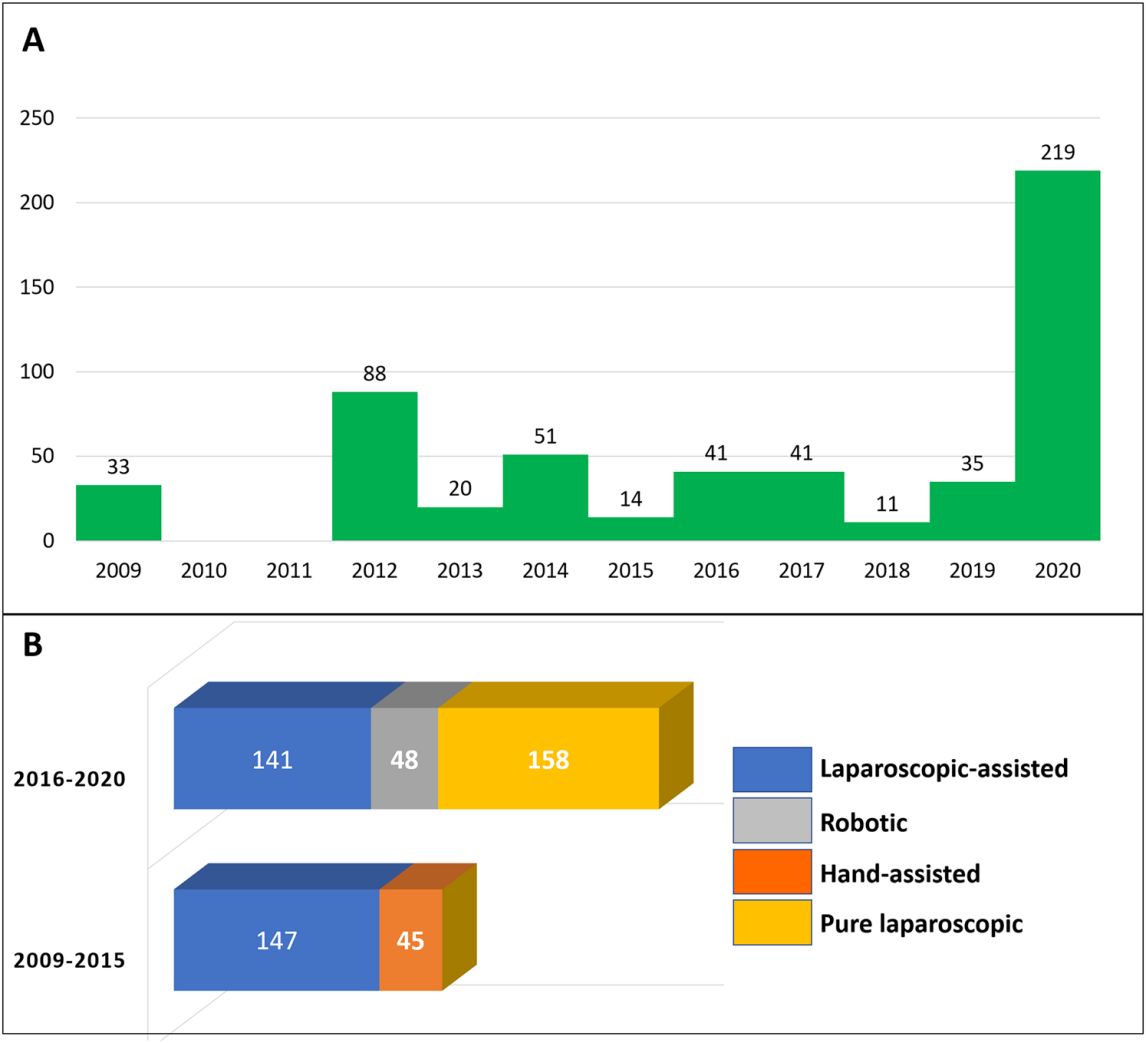
**A** Trend of published minimally invasive cases. **B** Different mini-invasive approaches in the different eras

A progressive numerical increment of the MILR cases was reported across the years (Fig. 3A). Contextually, a change in the different MILR techniques adopted was observed during the years (Fig. 3B). During the period 2009–2015, only laparoscopy-assisted and hand-assisted cases were reported. Contrarily, the pure laparoscopy and robotic cases were observed only during the period 2016–2020.

Data on conversion from minimally invasive to open approach were reported in 12 articles (*n* = 454). A total of 10/454 (2.2%) conversions were observed. Most of the conversions (7/10 cases) occurred during a pure laparoscopic approach, while the conversion occurred in three cases during a laparoscopic-assisted approach. In detail, the conversion during a pure laparoscopic approach was seen in 7/158 (4.4%) cases, followed by 3/290 (1.0%) donors approached with a laparoscopic-assisted technique. No conversions were reported in the 48 and 20 donors handled with a robotic or hand-assisted approach, respectively.

### Donor characteristics

The differences between the MILR and OLR group in terms of donor characteristics were reported in Table 2. As for the donor age, the MILR group showed a younger population (*P* value = 0.008). Regarding the donor sex, more males were observed in the MILR group (*P* value = 0.027). No differences were reported in terms of BMI value between the two groups (*P* value = 0.472).

### Postoperative course

Table 3 reported the postoperative course of the donors. As expected, a shorter operative duration was required in the OLR cases (*P* value = 0.020).

No other significant differences were observed between the MILR and OLR cases. Similar results were observed also when the estimated blood loss (*P* value = 0.384) and the length of hospital stay (*P* value = 0.972) were investigated.

### Complications after right hepatectomy

The number of donors experiencing a complication was clearly detailed in 14 studies (Table 3). In fact, in the study written by Choi et al. [21], only the total number of complications was reported instead of the number of donors experiencing a complication. Therefore, this study was not included for this meta-analysis.

**Fig. 4:**
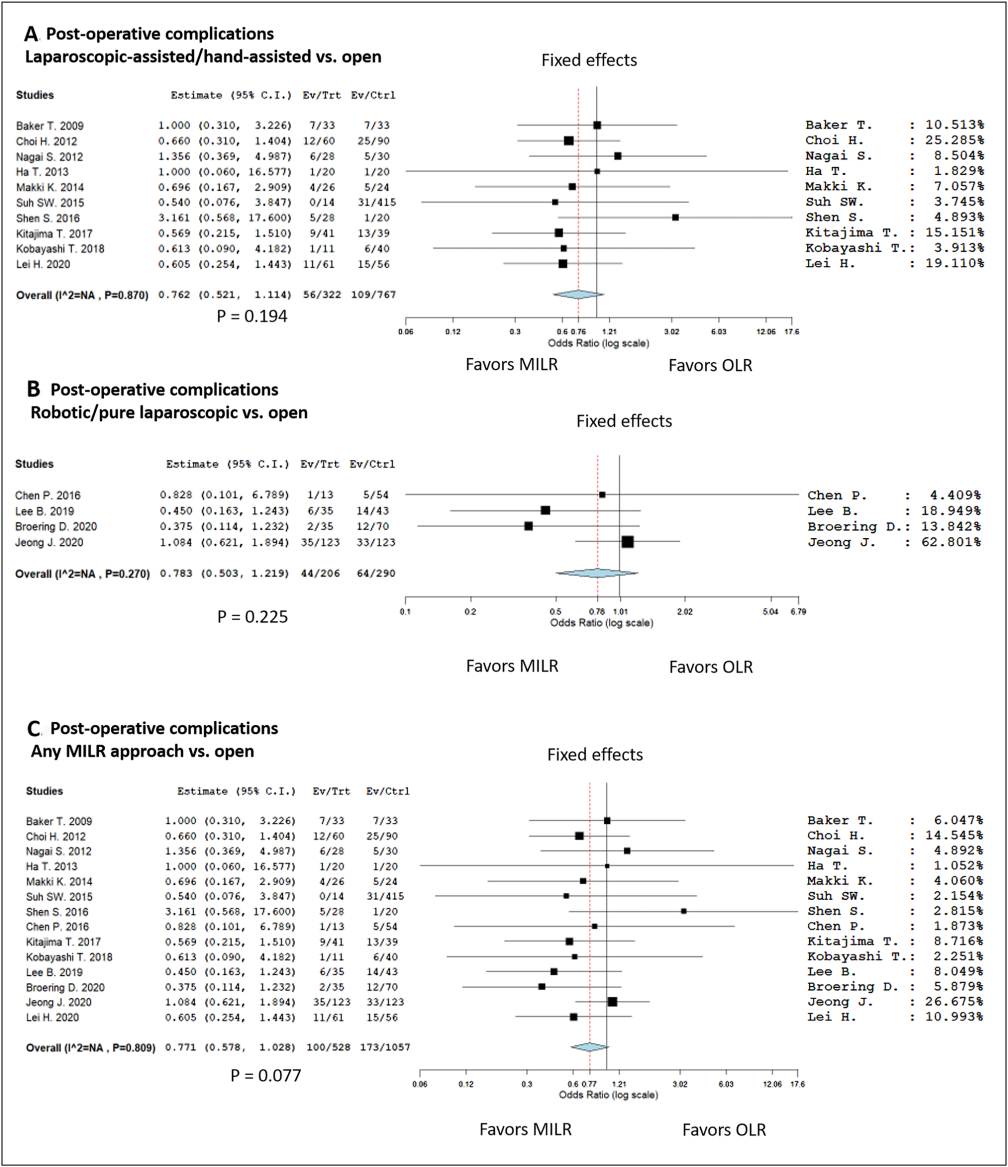
Forest plots and meta-analyses on the appearance of any complication grade according to Dindo-Clavien: **A** hybrid (laparoscopic-assisted and hand-assisted) vs. open; **B** pure laparoscopic (robotic and pure laparoscopic) vs. open; **C** all MILR techniques vs. open

A total of 100/528 MILR (18.9%) and 173/1057 OLR (16.4%) cases presented any grade of complication after donation. No statistical difference was reported between the two groups, with the MILR group only merging the statistical relevance in terms of advantage in terms of a lower number of complications (OR = 0.771, 95% CI 0.578–1.028; *P* value = 0.077) (Fig. 4). This datum was confirmed investigating separately the two sub-groups of MILR patients receiving a “hybrid” (laparoscopic-assisted and hand-assisted) or “pure laparoscopic” (robotic and pure laparoscopic) of MILR (Fig. 4).

**Fig. 5:**
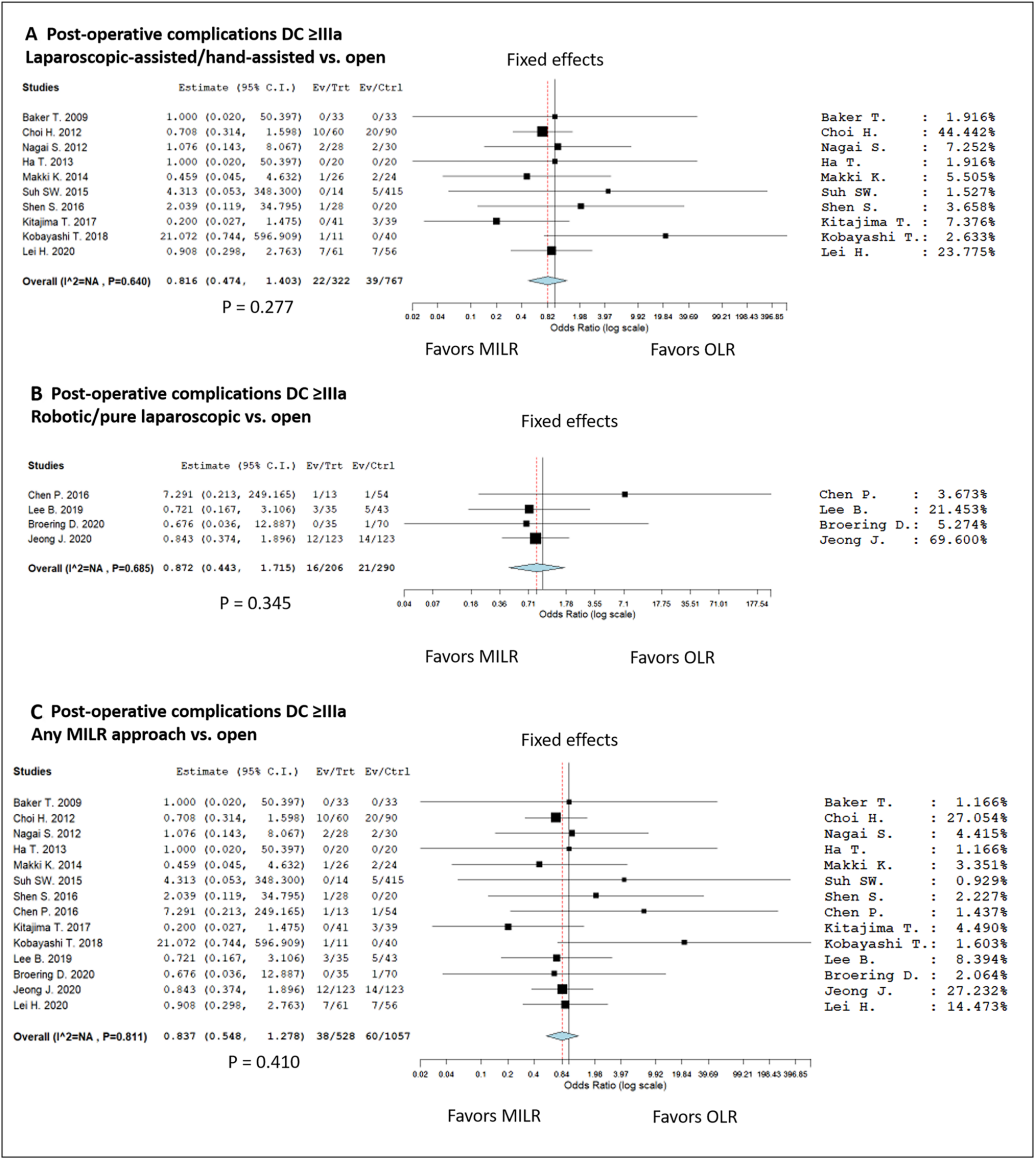
Forest plots and meta-analyses on the appearance of complications ≥ IIIa according to Dindo-Clavien: **A** hybrid (laparoscopic-assisted and hand-assisted) vs. open; **B** pure laparoscopic (robotic and pure laparoscopic) vs. open; **C** all MILR techniques vs. open

Only investigating the complications ≥ IIIa according to the Dindo-Clavien classification, 38/528 MILR (7.2%) and 60/1057 OLR (5.7%) cases were reported (Table 3). Importantly, no grade IV or V cases were observed in all the reported series. Also in this case, no statistical difference was reported between the two groups (OR = 0.837, 95% CI 0.578–1.278; *P* value = 0.401) (Fig. 5). This datum was further confirmed by investigating separately the two sub-groups of MILR patients receiving a “hybrid” (laparoscopic-assisted and hand-assisted) or “pure laparoscopic” (robotic and pure laparoscopic) of MILR (Fig. 5).

## Discussion

MILR has been introduced into clinical practice in the setting of living donation to reduce the potential risks of morbidity and mortality for the donor. As clearly reported in several studies, MILR consents to achieve several results when compared with OLR: (a) to minimize tissue trauma, (b) to reduce postoperative pain, (c) to achieve better aesthetic results by improving the psychosocial outcome of the donor, and (d) to allow a faster postoperative recovery and an early return to normal daily activities [8–10].

In the setting of left lateral sectionectomy as part of an adult-to-child live donation, the benefits of MILR have been largely explored [31, 32]. As a further confirmation of this evidence, the recent guidelines published on MILR and liver donation stated that “pure laparoscopic” donor hepatectomy is applicable to left lateral sectionectomy and should be considered standard practice once the team has fulfilled the adequate learning” [11].

On the opposite, more controversial results exist on the benefits of MILR for right hepatectomy in the setting of A2ALDLT. Conflicting results exist even in the meta-analyses already published on this topic [33–35].

Berardi reported in a meta-analysis (number of studies = 6; MILR = 171 vs. OLR = 223) that mini-invasive and open cases had similar results in terms of blood loss rates (*P* value = 0.45), operative time (*P* value = 0.45), and overall donor morbidity (*P* value = 0.86). On the opposite, the hospital stay duration was shorter in the MILR group (*P* value = 0.30) [33].

Zhang et al. [34] published another meta-analysis (number of studies = 7; MILR = 187 vs. OLR = 499) in which the mini-invasive approach reduced the intraoperative blood loss rates (*P* value = 0.002). However, no significant differences were reported in postoperative complications (*P* value = 0.80) and length of hospital stay (*P* value = 0.35).

Lastly, a meta-analysis published by Li et al. [35] (7 studies; MILR = 220 vs. OLR = 709) showed no difference in terms of post-operative complications (*P* value = 0.21), surgery duration (*P* value = 0.43), and blood loss rates (*P* value = 0.96).

In the present study, similar results were observed. No clear differences were reported in the postoperative clinical course when MILR and OLR cases of right hepatectomy for A2ALDLT were compared. As previously reported in the other meta-analyses [33–35] the MILR and OLR cases showed similar blood losses (*P* value = 0.38), AST and ALT peaks after surgery (*P* value = 0.09 and 0.08, respectively), postoperative complications (*P* value = 0.08), complications ≥ IIIa (*P* value = 0.40), and lengths of hospital stay (*P* value = 0.97).

The only significant datum was the longer duration of the MILR with respect to the open approach (*P* = 0.02), being this latter evidence not completely surprising when complex laparoscopic approaches are compared with open ones, mainly at the beginning of the learning curve [36].

With respect to the previously reported meta-analyses, the present study has the great benefit of investigating a larger number of cases (15 studies, *n* = 2094), with a total of 553 MILR cases, the largest even explored so far.

Such a beneficial numerical effect has also been corroborated by the possibility of investigating different mini-invasive approaches. Consequently, we were able to perform, for the first time, specific sub-analyses exploring separately only the hybrid (laparoscopy-assisted and hand-assisted) and the “pure laparoscopic” types of MILR (robotic and pure laparoscopy).

Thanks to these separate analyses, further investigation was given to the impact of MILR vs. OLR concerning the postoperative complications. However, the sub-analyses focused on “pure laparoscopy” approaches only further failed to show any difference between MILR and OLR. If we consider the cut-off of 50 procedures proposed by Rhu et al. for surpassing the learning curve [36], only a limited number of series was able to overpass it [17, 29, 30]. Approximately 40% of the reported MILR cases have been published only during 2020, and the pure laparoscopic approaches started to be published only in the last 4 years. Therefore, we can postulate that the complications reported in the present analysis should be affected by the surgeons' relatively short learning curve. In many cases, the reported studies investigate the first “laparoscopic” series of experienced “open” centers [11]. For this reason, the recent already cited international guidelines strongly recommend considering the laparoscopic approach as “applicable to selected right liver grafts”. We can only postulate that the growing experience in MILR will consent to observe a progressive reduction of complications in the next future.

 Unfortunately, some critical issues were impossible to be investigated in the present study. As an example, is strongly recommended in the international guideline, “large grafts and deviation from standard biliary and vascular anatomy may increase the difficulty of MILR procedure in right-lobe donation” [11]. These critical elements were impossible to be explored between the MILR and OLR groups, therefore adding a potential selection bias when the mini-invasive or open approach was decided. As a potential confirmation of a sort of selection of “easier” cases using the laparoscopic approach, we reported in our meta-analysis that younger cases were present in the MILR group (*P* value = 0.008).

Furthermore, one additional potential indirect observation comes from the result that more men were observed in the MILR group (*P* value = 0.027). It has been suggested that women more frequently present variations of biliary anatomy, making more challenging a MILR in a patient with such an anatomical condition [37, 38].

Another important aspect requiring further investigations is the role of the robotic approach. Only two studies reported robotic series [24, 28], limiting our possibility of further exploring the role of robotic surgery in this field. Lastly, relevant aspects like the use of different surgical instruments, the use of the Pringle Maneuver, and the vascular/biliary division methods need further studies to explain better their impact in the setting of MILR A2ALDLT donation.

In conclusion, MILR represents a novel and promising approach for improving adult-to-adult liver live donors' results. However, this surgery still pays the fee of having been only recently introduced. Therefore, no benefits have been reported regarding blood loss, length of stay, and postoperative complications. More extensive experiences are needed to re-evaluate the impact of MILR in right lobe live donation.

## Abbreviations

A2ALDLT: Adult-to-adult living-donor liver transplantation
CI: Confidence intervals
I2: Higgins statistic squared
LDLT: Living-donor liver transplantation
MILR: Minimally invasive liver resection
OLR: Open liver resection
OR: Odds Ratio
PRISMA: Preferred Reporting Items for Systemic Reviews and Meta-Analysis
WMD: Weighted mean difference

## References

[1] Raia S, Nery JR, Mies S (1989). Liver transplantation from live donors. Lancet.

[2] Strong RW, Lynch SV, Ong TH, Matsunami H, Koido Y, Balderson GA (1990). Successful liver transplantation from 4 living donor to her son. N Engl J Med.

[3] Cheah YL, Simpson MA, Pomposelli JJ, Pomfret EA (2013). Incidence of death and potentially life-threatening near-miss events in living donor hepatic lobectomy: a world-wide survey. Liver Transpl.

[4] Nudeshima J (1991). Obstacles to brain death and organ transplantation in Japan. Lancet.

[5] Hashikura Y, Makuuchi M, Kawasaki S, Matsunami H, Ikegami T, Nakazawa Y, Kiyosawa K, Ichida T (1994). Successful living-related partial liver transplantation to an adult patient. Lancet.

[6] Lo CM, Fan ST, Liu CL, Wei WI, Lo RJ, Lai CL, Chan JK, Ng IO, Fung A, Wong J (1997). Adult-to-adult living donor liver transplantation using extended right lobe grafts. Ann Surg.

[7] Cronin DC, Millis JM, Siegler M (2001). Transplantation of liver grafts from living donors into adults—too much, too soon. N Engl J Med.

[8] Buell JF, Cherqui D, Geller DA, O'Rourke N, Iannitti D, Dagher I, Koffron AJ, Thomas M, Gayet B, Han HS, Wakabayashi G, Belli G, Kaneko H, Ker CG, Scatton O, Laurent A, Abdalla EK, Chaudhury P, Dutson E, Gamblin C, D'Angelica M, Nagorney D, Testa G, Labow D, Manas D, Poon RT, Nelson H, Martin R, Clary B, Pinson WC, Martinie J, Vauthey JN, Goldstein R, Roayaie S, Barlet D, Espat J, Abecassis M, Rees M, Fong Y, McMasters KM, Broelsch C, Busuttil R, Belghiti J, Strasberg S, Chari RS, World Consensus Conference on Laparoscopic Surgery (2009). The international position on laparoscopic liver surgery: The Louisville Statement, 2008. Ann Surg.

[9] Wakabayashi G, Cherqui D, Geller DA, Buell JF, Kaneko H, Han HS, Asbun H, O’Rourke N, Tanabe M, Koffron AJ, Tsung A, Soubrane O, Machado MA, Gayet B, Troisi RI, Pessaux P, Van Dam RM, Scatton O, Hilal MA, Belli G, Kwon CH, Edwin B, Choi GH, Aldrighetti LA, Cai X, Cleary S, Chen KH, Schön MR, Sugioka A, Tang CN, Herman P, Pekolj J, Chen XP, Dagher I, Jarnagin W, Yamamoto M, Strong R, Jagannath P, Lo CM, Clavien PA, Kokudo N, Barkun J, Strasberg SM (2015). Recommendations for laparoscopic liver resection: a report from the second international consensus conference held in Morioka. Ann Surg.

[10] Abu Hilal M, Aldrighetti L, Dagher I, Edwin B, Troisi RI, Alikhanov R, Aroori S, Belli G, Besselink M, Briceno J, Gayet B, D’Hondt M, Lesurtel M, Menon K, Lodge P, Rotellar F, Santoyo J, Scatton O, Soubrane O, Sutcliffe R, Van Dam R, White S, Halls MC, Cipriani F, Van der Poel M, Ciria R, Barkhatov L, Gomez-Luque Y, Ocana-Garcia S, Cook A, Buell J, Clavien PA, Dervenis C, Fusai G, Geller D, Lang H, Primrose J, Taylor M, Van Gulik T, Wakabayashi G, Asbun H, Cherqui D (2018). The Southampton Consensus Guidelines for laparoscopic liver surgery: from indication to implementation. Ann Surg.

[11] Cherqui D, Ciria R, Kwon CHD, Kim KH, Broering D, Wakabayashi G, Samstein B, Troisi RI, Han HS, Rotellar F, Soubrane O, Briceño J, Alconchel F, Ayllón MD, Berardi G, Cauchy F, Luque IG, Hong SK, Yoon YY, Egawa H, Lerut J, Lo CM, Rela M, Sapisochin G, Suh KS (2021). Expert Consensus Guidelines on minimally invasive donor hepatectomy for living donor liver transplantation from innovation to implementation: A Joint Initiative From the International Laparoscopic Liver Society (ILLS) and the Asian-Pacific Hepato-Pancreato-Biliary Association (A-PHPBA). Ann Surg.

[12] Beller EM, Glasziou PP, Altman DG (2013). PRISMA for abstracts: Reporting systematic reviews in journal and conference abstracts. PLoS Med.

[13] Sterne JA, Hernán MA, Reeves BC, Savović J, Berkman ND, Viswanathan M, Henry D, Altman DG, Ansari MT, Boutron I, Carpenter JR, Chan AW, Churchill R, Deeks JJ, Hróbjartsson A, Kirkham J, Jüni P, Loke YK, Pigott TD, Ramsay CR, Regidor D, Rothstein HR, Sandhu L, Santaguida PL, Schünemann HJ, Shea B, Shrier I, Tugwell P, Turner L, Valentine JC, Waddington H, Waters E, Wells GA, Whiting PF, Higgins JP (2016). ROBINS-I: a tool for assessing risk of bias in non-randomised studies of interventions. BMJ.

[14] Wallace BC, Dahabreh IJ, Trikalinos TA, Lau J, Trow P, Schmid CH (2012). Losing the gap between methodologists and end-users: R as a computational back-end. J Stat Softw.

[15] McGrath S, Zhao XF, Steele R, Thombs BD, Benedetti A, The DEPRESsion Screening Data (DEPRESSD) Collaboration (2020). Estimating the sample mean and standard deviation from commonly reported quantiles in meta-analysis. Stat Methods Med Res.

[16] Baker TB, Jay CL, Ladner DP, Preczewski LB, Clark L, Holl J, Abecassis MM (2009). Laparoscopy-assisted and open living donor right hepatectomy: a comparative study of outcomes. Surgery.

[17] Choi HJ, You YK, Na GH, Hong TH, Shetty GS, Kim DG (2012). Single-port laparoscopy-assisted donor right hepatectomy in living donor liver transplantation: sensible approach or unnecessary hindrance?. Transpl Proceed.

[18] Nagai S, Brown L, Yoshida A, Kim D, Kazimi M, Abouljoud MS (2012). Mini-incision right hepatic lobectomy with or without laparoscopic assistance for living donor hepatectomy. Liver Transpl.

[19] Ha TY, Hwang S, Ahn CS, Kim KH, Moon DB, Song GW, Jung DH, Park GC, Namgoong JM, Park CS, Park YH, Park HW, Kang SH, Jung BH, Lee SG (2013). Role of hand-assisted laparoscopic surgery in living-donor right liver harvest. Transpl Proceed.

[20] Makki K, Chorasiya VK, Sood G, Srivastava PK, Dargan P, Vij V (2014). Laparoscopy-assisted hepatectomy versus conventional (open) hepatectomy for living donors: when you know better, you do better. Liver Transpl.

[21] Choi YR, Yi NJ, Lee KW, Suh KS (2014). Laparoscopic and minimal incisional donor hepatectomy. Transplantation.

[22] Suh SW, Lee KW, Lee JM, Choi YR, Yi NJ, Suh KS (2015). Clinical outcomes of and patient satisfaction with different incision methods for donor hepatectomy in living donor liver transplantation. Liver Transpl.

[23] Shen S, Zhang W, Jiang L, Yan L, Yang J (2016). Comparison of upper midline incision with and without laparoscopic assistance for living-donor right hepatectomy. Transpl Proceed.

[24] Chen PD, Wu CY, Hu RH, Ho CM, Lee PH, Lai HS, Lin MT, Wu YM (2016). Robotic liver donor right hepatectomy—a pure, minimally invasive approach. Liver Transpl.

[25] Kitajima T, Kaido T, Iida T, Seo S, Taura K, Fujimoto Y, Ogawa K, Hatano E, Okajima H, Uemoto S (2017). Short-term outcomes of laparoscopy-assisted hybrid living donor hepatectomy: a comparison with the conventional open procedure. Surg Endosc.

[26] Kobayashi T, Miura K, Ishikawa H, Soma D, Ando T, Yuza K, Hirose Y, Katada T, Takizawa K, Nagahashi M, Sakata J, Kameyama H, Wakai T (2018). Long-term follow-up of laparoscope-assisted living donor hepatectomy. Transpl Proceed.

[27] Lee B, Choi Y, Han HS, Yoon YS, Cho JY, Kim S, Kim KH, Hyun IG (2019). Comparison of pure laparoscopic and open living donor right hepatectomy after a learning curve. Clin Transplant.

[28] Broering DC, Elsheikh Y, Alnemary Y, Zidan A, Elsarawy A, Saleh Y, Alabbad S, Sturdevant M, Wu YM, Troisi RI (2020). Robotic versus open right lobe donor hepatectomy for adult living donor liver transplantation: a propensity score-matched analysis. Liver Transpl.

[29] Jeong JS, Wi W, Chung YJ, Kim JM, Choi GS, Kwon CHD, Han S, Gwak MS, Kim GS, Ko JS (2020). Comparison of perioperative outcomes between pure laparoscopic surgery and open right hepatectomy in living donor hepatectomy: propensity score matching analysis. Sci Rep.

[30] Lei HJ, Lin NC, Chen CY, Chou SC, Chung MH, Shyr BU, Tsai HL, Hsia CY, Liu CS, Loong CC (2020). Safe strategy to initiate total laparoscopic donor right hepatectomy: a stepwise approach from a laparoscopy-assisted method. World J Surg.

[31] Almodhaiberi H, Kim SH, Kim KH (2018). Totally laparoscopic living donor left hepatectomy for liver transplantation in a child. Surg Endosc.

[32] Park JI, Kim KH, Lee SG (2015). Laparoscopic living donor hepatectomy: a review of current status. J Hepatobiliary Pancreat Sci.

[33] Berardi G, Tomassini F, Troisi RI (2015). Comparison between minimally invasive and open living donor hepatectomy: a systematic review and meta-analysis. Liver Transpl.

[34] Zhang B, Pan Y, Chen K, Maher H, Chen MY, Zhu HP, Zhu YB, Dai Y, Chen J, Cai XJ (2017). Laparoscopy-assisted versus open hepatectomy for live liver donor: systematic review and meta-analysis. Can J Gastroenterol Hepatol.

[35] Li H, Zhang JB, Chen XL, Fan L, Wang L, Li SH, Zheng QL, Wang XM, Yang Y, Chen GH, Wang GS (2017). Different techniques for harvesting grafts for living donor liver transplantation: a systematic review and meta-analysis. World J Gastroenterol.

[36] Rhu J, Choi GS, Kwon CHD, Kim JM, Joh JW (2020). Learning curve of laparoscopic living donor right hepatectomy. Br J Surg.

[37] Uysal F, Obuz F, Uçar A, Seçil M, Igci E, Dicle O (2014). Anatomic variations of the intrahepatic bile ducts: analysis of magnetic resonance cholangiopancreatography in 1011 consecutive patients. Digestion.

[38] Cucchetti A, Peri E, Cescon M, Zanello M, Ercolani G, Zanfi C, Bertuzzo V, Di Gioia P, Pinna AD (2011). Anatomic variations of intrahepatic bile ducts in a European series and meta-analysis of the literature. J Gastrointest Surg.

